# Supplementary material for: Modeling glioblastoma heterogeneity as a dynamic network of cell states
Source: Mol Syst Biol. 2021 Sep 16;17(9):e10105. doi: 10.15252/msb.202010105 (PMC8444284; doi:10.15252/msb.202010105)
Supplement: Supplementary file 5 — Source Data for Figure 3 [file MSB-17-e10105-s001.zip › Figure3A_sourcedata/GSEA_3065/nosigdb_state5.GseaPreranked.1622718435914/RICHARDS_NOWAKOWSKI_SCIENCE_2017_MGE_IPC3_UPREG.html]

Details for gene set RICHARDS\_NOWAKOWSKI\_SCIENCE\_2017\_MGE\_IPC3\_UPREG[GSEA]

|  || Dataset | state5 |
| Phenotype | NoPhenotypeAvailable |
| Upregulated in class | na\_neg |
| GeneSet | RICHARDS\_NOWAKOWSKI\_SCIENCE\_2017\_MGE\_IPC3\_UPREG |
| Enrichment Score (ES) | -0.5299764 |
| Normalized Enrichment Score (NES) | -1.8634373 |
| Nominal p-value | 0.0 |
| FDR q-value | 8.6926675E-4 |
| FWER p-Value | 0.019 |
Table: GSEA Results Summary

  

Fig 1: Enrichment plot: RICHARDS\_NOWAKOWSKI\_SCIENCE\_2017\_MGE\_IPC3\_UPREG      
 Profile of the Running ES Score & Positions of GeneSet Members on the Rank Ordered List

  

| PROBE | GENE SYMBOL | GENE\_TITLE | RANK IN GENE LIST | RANK METRIC SCORE | RUNNING ES | CORE ENRICHMENT || 1 | SOX6 |  |  | 28 | 0.754 | 0.0198 | No |
| 2 | HSPA5 |  |  | 43 | 0.698 | 0.0395 | No |
| 3 | MTMR4 |  |  | 108 | 0.518 | 0.0481 | No |
| 4 | DLL3 |  |  | 171 | 0.463 | 0.0552 | No |
| 5 | HELLS |  |  | 280 | 0.393 | 0.0550 | No |
| 6 | CHD7 |  |  | 312 | 0.376 | 0.0630 | No |
| 7 | NUCB2 |  |  | 332 | 0.367 | 0.0720 | No |
| 8 | SERINC5 |  |  | 477 | 0.318 | 0.0655 | No |
| 9 | CDCA7 |  |  | 495 | 0.313 | 0.0731 | No |
| 10 | MSI2 |  |  | 496 | 0.313 | 0.0826 | No |
| 11 | EPRS |  |  | 506 | 0.309 | 0.0910 | No |
| 12 | ST8SIA5 |  |  | 583 | 0.290 | 0.0913 | No |
| 13 | HES6 |  |  | 653 | 0.274 | 0.0919 | No |
| 14 | HNRNPH1 |  |  | 736 | 0.253 | 0.0903 | No |
| 15 | SREBF2 |  |  | 882 | 0.228 | 0.0809 | No |
| 16 | RAB3IP |  |  | 883 | 0.228 | 0.0879 | No |
| 17 | ILDR2 |  |  | 965 | 0.215 | 0.0853 | No |
| 18 | BAZ2B |  |  | 974 | 0.214 | 0.0909 | No |
| 19 | GLCCI1 |  |  | 1172 | 0.188 | 0.0745 | No |
| 20 | CERS6 |  |  | 1208 | 0.183 | 0.0761 | No |
| 21 | TMEM123 |  |  | 1293 | 0.172 | 0.0719 | No |
| 22 | CASD1 |  |  | 1424 | 0.160 | 0.0621 | No |
| 23 | CENPF |  |  | 1531 | 0.149 | 0.0547 | No |
| 24 | EPHA4 |  |  | 1574 | 0.143 | 0.0543 | No |
| 25 | CCDC88A |  |  | 1656 | 0.137 | 0.0494 | No |
| 26 | RBL1 |  |  | 1669 | 0.136 | 0.0522 | No |
| 27 | DCLK2 |  |  | 1694 | 0.134 | 0.0535 | No |
| 28 | SACS |  |  | 1773 | 0.128 | 0.0486 | No |
| 29 | DYRK1A |  |  | 1812 | 0.126 | 0.0482 | No |
| 30 | HNRNPA2B1 |  |  | 1852 | 0.122 | 0.0475 | No |
| 31 | RFTN2 |  |  | 1867 | 0.121 | 0.0496 | No |
| 32 | MAP1A |  |  | 1958 | 0.114 | 0.0430 | No |
| 33 | NOP56 |  |  | 2390 | 0.086 | -0.0030 | No |
| 34 | NES |  |  | 2417 | 0.084 | -0.0034 | No |
| 35 | HAT1 |  |  | 2459 | 0.082 | -0.0055 | No |
| 36 | TEAD1 |  |  | 2525 | 0.077 | -0.0104 | No |
| 37 | ABAT |  |  | 3020 | 0.051 | -0.0646 | No |
| 38 | CORO1C |  |  | 3217 | 0.040 | -0.0855 | No |
| 39 | TPR |  |  | 3290 | 0.037 | -0.0924 | No |
| 40 | MTAP |  |  | 3351 | 0.034 | -0.0982 | No |
| 41 | CPSF7 |  |  | 3399 | 0.032 | -0.1025 | No |
| 42 | WDR70 |  |  | 3480 | 0.028 | -0.1107 | No |
| 43 | UPF3B |  |  | 3527 | 0.026 | -0.1151 | No |
| 44 | CEP83 |  |  | 3941 | 0.010 | -0.1614 | No |
| 45 | CHD3 |  |  | 4029 | 0.006 | -0.1710 | No |
| 46 | PBX3 |  |  | 4054 | 0.005 | -0.1735 | No |
| 47 | FEM1C |  |  | 4142 | 0.001 | -0.1833 | No |
| 48 | CCNE2 |  |  | 4180 | -0.001 | -0.1875 | No |
| 49 | SOX2 |  |  | 4271 | -0.005 | -0.1975 | No |
| 50 | CCND2 |  |  | 4539 | -0.016 | -0.2271 | No |
| 51 | SCYL2 |  |  | 4584 | -0.017 | -0.2315 | No |
| 52 | MCM2 |  |  | 4824 | -0.027 | -0.2577 | No |
| 53 | KLHL13 |  |  | 4935 | -0.031 | -0.2691 | No |
| 54 | GINS2 |  |  | 5024 | -0.034 | -0.2780 | No |
| 55 | INSM1 |  |  | 5071 | -0.036 | -0.2821 | No |
| 56 | TCF3 |  |  | 5402 | -0.048 | -0.3178 | No |
| 57 | FBXW7 |  |  | 5435 | -0.049 | -0.3199 | No |
| 58 | DCTN4 |  |  | 5560 | -0.055 | -0.3322 | No |
| 59 | OXR1 |  |  | 5592 | -0.057 | -0.3340 | No |
| 60 | CLSPN |  |  | 5614 | -0.058 | -0.3346 | No |
| 61 | RND3 |  |  | 5668 | -0.061 | -0.3387 | No |
| 62 | RNASEH2B |  |  | 5954 | -0.072 | -0.3686 | No |
| 63 | BAZ1A |  |  | 6008 | -0.074 | -0.3723 | No |
| 64 | MAPRE2 |  |  | 6079 | -0.078 | -0.3778 | No |
| 65 | CS |  |  | 6219 | -0.086 | -0.3909 | No |
| 66 | RGS16 |  |  | 6543 | -0.104 | -0.4241 | No |
| 67 | ANP32E |  |  | 6655 | -0.111 | -0.4332 | No |
| 68 | XPO6 |  |  | 6675 | -0.112 | -0.4320 | No |
| 69 | PPCS |  |  | 6716 | -0.115 | -0.4330 | No |
| 70 | ICE1 |  |  | 6861 | -0.126 | -0.4454 | No |
| 71 | PDCL |  |  | 6875 | -0.128 | -0.4429 | No |
| 72 | CDC42SE2 |  |  | 6886 | -0.128 | -0.4402 | No |
| 73 | SF3B4 |  |  | 6929 | -0.131 | -0.4409 | No |
| 74 | VEZF1 |  |  | 7040 | -0.140 | -0.4490 | No |
| 75 | CITED2 |  |  | 7120 | -0.147 | -0.4534 | No |
| 76 | PTPRN2 |  |  | 7125 | -0.148 | -0.4494 | No |
| 77 | TMPO |  |  | 7296 | -0.167 | -0.4635 | No |
| 78 | TOX3 |  |  | 7342 | -0.173 | -0.4633 | No |
| 79 | USP1 |  |  | 7398 | -0.181 | -0.4640 | No |
| 80 | SOX4 |  |  | 7958 | -0.268 | -0.5188 | No |
| 81 | HMGB2 |  |  | 7973 | -0.271 | -0.5122 | No |
| 82 | TSG101 |  |  | 7995 | -0.275 | -0.5062 | No |
| 83 | CDK6 |  |  | 8156 | -0.310 | -0.5148 | No |
| 84 | PSMB2 |  |  | 8292 | -0.352 | -0.5192 | Yes |
| 85 | EIF1AX |  |  | 8331 | -0.363 | -0.5125 | Yes |
| 86 | OLA1 |  |  | 8341 | -0.367 | -0.5023 | Yes |
| 87 | NUDT1 |  |  | 8359 | -0.373 | -0.4928 | Yes |
| 88 | SH3BGRL |  |  | 8409 | -0.390 | -0.4865 | Yes |
| 89 | HNRNPC |  |  | 8413 | -0.392 | -0.4749 | Yes |
| 90 | CKB |  |  | 8448 | -0.407 | -0.4663 | Yes |
| 91 | H1FX |  |  | 8466 | -0.417 | -0.4555 | Yes |
| 92 | PSMC2 |  |  | 8497 | -0.433 | -0.4457 | Yes |
| 93 | PFN2 |  |  | 8545 | -0.455 | -0.4371 | Yes |
| 94 | HNRNPA1 |  |  | 8570 | -0.469 | -0.4255 | Yes |
| 95 | HMGN2 |  |  | 8608 | -0.496 | -0.4145 | Yes |
| 96 | NDUFC1 |  |  | 8616 | -0.503 | -0.4000 | Yes |
| 97 | HMGB1 |  |  | 8653 | -0.527 | -0.3880 | Yes |
| 98 | MARCKS |  |  | 8667 | -0.537 | -0.3731 | Yes |
| 99 | UBB |  |  | 8745 | -0.602 | -0.3634 | Yes |
| 100 | STMN1 |  |  | 8783 | -0.638 | -0.3481 | Yes |
| 101 | RPL21 |  |  | 8802 | -0.655 | -0.3302 | Yes |
| 102 | MZT2B |  |  | 8816 | -0.665 | -0.3114 | Yes |
| 103 | RPS11 |  |  | 8830 | -0.687 | -0.2919 | Yes |
| 104 | SUMO2 |  |  | 8852 | -0.712 | -0.2726 | Yes |
| 105 | ACTG1 |  |  | 8859 | -0.718 | -0.2514 | Yes |
| 106 | GNG5 |  |  | 8866 | -0.728 | -0.2299 | Yes |
| 107 | H3F3A |  |  | 8867 | -0.728 | -0.2077 | Yes |
| 108 | PTMA |  |  | 8894 | -0.764 | -0.1873 | Yes |
| 109 | ACTB |  |  | 8912 | -0.793 | -0.1651 | Yes |
| 110 | SERF2 |  |  | 8920 | -0.816 | -0.1410 | Yes |
| 111 | RPL23A |  |  | 8934 | -0.845 | -0.1167 | Yes |
| 112 | YBX1 |  |  | 8948 | -0.872 | -0.0916 | Yes |
| 113 | RPL4 |  |  | 8950 | -0.881 | -0.0648 | Yes |
| 114 | TUBA1B |  |  | 8980 | -1.103 | -0.0345 | Yes |
| 115 | TMSB4X |  |  | 8983 | -1.142 | 0.0001 | Yes |
Table: GSEA details [plain text format]

  

Fig 2: RICHARDS\_NOWAKOWSKI\_SCIENCE\_2017\_MGE\_IPC3\_UPREG: Random ES distribution      
 Gene set null distribution of ES for **RICHARDS\_NOWAKOWSKI\_SCIENCE\_2017\_MGE\_IPC3\_UPREG**
